# A novel *TBX19* gene mutation in patients with isolated ACTH deficiency from distinct families with a common geographical origin

**DOI:** 10.3389/fendo.2022.1080649

**Published:** 2023-02-15

**Authors:** Théo Charnay, Gregory Mougel, Cyril Amouroux, Iva Gueorguieva, Florence Joubert, Morgane Pertuit, Rachel Reynaud, Anne Barlier, Thierry Brue, Alexandru Saveanu

**Affiliations:** ^1^ Institut National de la Santé et de la Recherche Médicale (INSERM) U1251, Marseille Medical Genetics (MMG), Institut Marseille Maladies Rares (MarMaRa), Aix-Marseille Université, Marseille, France; ^2^ Laboratory of Molecular Biology, Centre Hospitalier Universitaire Conception, Assistance Publique-Hôpitaux de Marseille (AP-HM), Marseille, France; ^3^ Paediatric Department, Endocrinology Unit, Arnaud de Villeneuve Hospital, Montpellier University Hospital, Montpellier, France; ^4^ Paediatric Department, Endocrinology Unit, Children’s Center, Jeanne-de-Flandre Hospital, Lille University Hospital, Lille, France; ^5^ Department of Pediatrics, Centre Hospitalier d’Avignon, Avignon, France; ^6^ Department of Multidisciplinary Peadiatrics, Centre de Référence des Maladies Rares d’origine hypophysaire HYPO, Hôpital Timone-Enfants, Assistance Publique-Hôpitaux de Marseille (AP-HM), Marseille, France; ^7^ Department of Endocrinology, Centre de Référence des Maladies Rares de l’hypophyse HYPO, Hôpital de la Conception, Assistance Publique-Hôpitaux de Marseille (AP-HM), Marseille, France

**Keywords:** isolated ACTH deficiency, low ACTH and cortisol, adrenal insufficiency, TBX19, TPIT, recessive disorder, founder effect

## Abstract

Isolated ACTH deficiency (IAD) is a life-threatening condition, particularly in the neonatal period, while a main consequence of undiagnosed isolated ACTH deficiency in survivors is cognitive impairment. *TBX19* is involved in the differentiation and proliferation of corticotropic cells and *TBX19* mutations are responsible for more than 60% of neonatal cases of IAD. We describe a new variant of the main *TBX19* transcript (NM 005149.3, c.840del (p.(Glu280Asp fs*27)), classified as pathogenic, whose pathogenicity is assumed to be due to nonsense mediated decay leading to non-expression of T-box transcription factor TBX19. Moreover we summarize the TBX19 mutations published as individual cases since our last large cohort. Interestingly, this pathogenic variant was identified in four patients from three apparently unrelated families. Two of these families were consanguineous, and after investigations all of three were discovered to have roots in the same mountainous region of northern Morocco, suggesting a founder effect. Early diagnosis, timely treatment (hydrocortisone therapy) and preventive education allowed normal development, growth and quality of life in all patients.

## 1 Introduction

Isolated ACTH deficiency (IAD) is a rare cause of adrenocortical insufficiency, characterized by low ACTH and cortisol levels, which causes early and severe secondary adrenal insufficiency without other pituitary defects. Low cortisol manifests in the neonatal period as hypoglycemia, prolonged cholestatic jaundice, and seizures (1, 2), and families frequently have a history of neonatal death. Only a few cases of delayed diagnosis at up to 1 year of age have been reported. *TBX19* mutations have been implicated in more than 60 percent of cases of neonatal IAD. As in other autosomal recessive diseases, incidence is much higher in consanguineous families. Since adrenal insufficiency is a life threatening condition, early detection of *TBX19* mutations is crucial to ensure rapid management at birth and avoid neonatal mortality.

The *TBX19* gene (formerly known as *TPIT*) codes for a T-box transcription factor found in proopiomelanocortin (POMC)-expressing pituitary cells (3). TBX19 is required for the terminal differentiation of these cells and the expression of the *POMC* gene. *TBX19* is located on chromosome 1q24.2 and its main transcript is composed of 8 exons (NM 005149.3) (4).

Since the description in 2012 of a series of 91 IAD patients with 21 pathogenic variants (1), 13 other mutations have been described in patients with IAD, mostly as individual cases, and six further mutations have been reported in public databases (ClinVar, LOVD, HGMD) (5–16). *TBX19* mutations are distributed across all exons other than exon 8 and all types of pathogenic variants have been described, including missense and truncating mutations (nonsense or indel frameshifts, larger deletions of one or several exons, or intronic mutations affecting splicing), in homozygous or compound heterozygous state.

We report four cases of IAD in three different families, all due to a new homozygous *TBX19* mutation, c.840del (p.(Glu280Asp fs*27)). In two of the families, the patient’s parents were consanguineous. All parents were carriers of the c.840del mutation in heterozygous state, and despite living in different regions of France, they all came from the same small area in northern Morocco.

## 2 Subjects and methods

### 2.1 Subjects

Patients with isolated ACTH deficiency (IAD) were screened for *TBX19* variants (RefSeq: NM 005149.3; OMIM 604614) in the context of GENHYPOPIT, a clinical research network created to investigate the genetic basis of congenital hypopituitarism (17, 18). Patients with a known postnatal cause of acquired IAD were excluded. Hormonal studies and intracranial imaging were performed in all patients in the respective hospitals. Pituitary and cerebral malformations were sought on MRI and recorded. Blood samples were collected from patients and, if possible, from first-degree relatives. Written informed consent was obtained from parents, caretakers, or guardians on behalf of children enrolled in the study. The study was approved by the ethics committee of the French Ministry of Health (*Cellule de Bioéthique, Direction générale de la Recherche et de l’Innovation*, approval n° DC 2019-3568) and by the French data protection authority (*Commission Nationale Informatique et Libertés*, CNIL 16 September 2016, N° 1991429 v 0).

### 2.2 Next-generation sequencing

Genomic DNA was extracted from blood lymphocytes (standard EDTA samples) using QiaSymphony DS DNA Midi kits (Qiagen, France). The 8 exons and 20 bp flanking introns of *TBX19* were amplified using QIAseq Targeted DNA Custom Panels (Qiagen, France). Other genes involved either in ACTH deficiency associated with common immune variable deficit (David syndrome - *NFKB2*) or associated to other pituitary deficits in non-syndromic combined pituitary deficiency (17) provided the necessary diversity for the interpretation of SNV (single nucleotide variant) and CNV (copy number variants) data. The library was prepared according to manufacturer instructions. All samples were then sequenced using a MiseqDX device (Miseq V2 Reagent Kit, 300 cycles; Illumina^®^, San Diego, CA, USA). Sequencing data were exported and further analyzed (base calling, read filtering, alignment to the human genome (GRCH37/hg19) and annotated using CLC Genomic Workbench v6 (Qiagen, France). Variant calling analysis was verified using VariantStudio v3.0 (Illumina^®^, San Diego, CA, USA). Sequence coverage was deeper than 30× across all targeted regions. All variants of interest were visually inspected in the Integrative Genomics Viewer (“https://www.broadinstitute.org/igv”) to evaluate mapping and variant calling quality. Candidate variants were classified as pathogenic, likely pathogenic, variants of uncertain significance (VUS), likely benign, or benign according to ACMG (American College of Medical Genetics and Genomics) guidelines (19). Variants classified as pathogenic, likely pathogenic, or VUS were confirmed by Sanger sequencing using specific primers for each exon (4). Sequencing was performed on an ABI 3500xl DxGenetic Analyzer (Thermo Fisher Scientific).

## 3 Clinical presentation

### 3.1 Family 1

The index case was a boy born at term with neonatal hypoglycemia and axial hypotonia, both of which resolved spontaneously. The patient had no other severe clinical symptoms until 5 months of age, when he developed hypoglycemia associated with prolonged cholestatic jaundice. Endocrine investigations revealed cortisol and ACTH levels below laboratory detection thresholds (*<*27 nmol/and <1.1 pmol/L, respectively). The patient recovered rapidly after hydrocortisone treatment was started (2.5 mg, three times daily). Other pituitary hormone levels were normal for age, with the exception of TSH, which was initially above the normal range (16.79 mUI/L versus 0.73–8.35 mUI/L) but rapidly normalized. The anterior and posterior pituitary and the pituitary stalk were normal on MRI. Replacement therapy was continued with no severe infectious or episodes of acute adrenal insufficiency and normal growth (99th percentile) as of last follow-up, at 3 years of age.

Targeted NGS sequencing revealed a homozygous c.840del mutation in *TBX19*. The patient’s parents, who live in southeastern France but were born in Morocco, are first cousins. Both are heterozygous carriers of the mutation and have no signs of ACTH deficiency. They have two other healthy children (not investigated) and the mother reported two miscarriages in the first trimester of pregnancy.

### 3.2 Family 2

The index case was a boy who presented respiratory distress with pulmonary arterial hypertension within minutes of birth, requiring intubation for 3 days and then noninvasive ventilation for 11 days. The patient also had hemodynamic instability (treated by intravenous fluids and noradrenaline), severe hypoglycemia, prolonged cholestatic jaundice, and axial hypotonia. Brain MRI and EEG findings were normal.

The patient was admitted to the emergency department two months later for weakness and loss of appetite in the context of severe bronchiolitis. Laboratory tests showed severe hypoglycemia (0.2 g/L), hyponatremia and hyperkalemia. Hormone testing revealed cortisol and ACTH levels below laboratory detection thresholds (*<*3 nmol/L and <0.6 pmol/L, respectively) leading to a diagnosis of ACTH deficiency. Aldosterone and renin levels were within normal ranges. All investigated pituitary axes and free thyroid hormones were in the normal range. Hydrocortisone replacement therapy was initiated (15 mg/m2/day) and the patient’s clinical condition improved rapidly. Subsequent pituitary MRI showed a moderately hypoplastic anterior pituitary. Clinical examination and echocardiography revealed a bicuspid aortic valve. Hydrocortisone therapy was continued at 15 mg/m2/day (increased as appropriate in stress situations). As of last follow-up, at 5 years of age, the patient was in good health, with no other severe infections or episodes of adrenal insufficiency. The episodes of severe hypoglycemia and hyponatremia in infancy seem not to have affected psychomotor development.

Targeted NGS sequencing revealed a homozygous c.840del mutation in *TBX19*. The patient’s parents, who live in the south of France but were born in Morocco, are first cousins. Both are asymptomatic heterozygous carriers of the *TBX19* mutation. No miscarriages were reported. They subsequently had a second child, who was also found to be a heterozygous carrier of the *TBX19* variant, and is also asymptomatic.

### 3.3 Family 3

The index case was a full-term girl who had repeated episodes of hypoglycemia (0.10 g/L) after 8 h of life, associated with low plasma cortisol and ACTH levels (below laboratory detection thresholds; *<*27 nmol/L and <1.1 pmol/L, respectively). Daily oral hydrocortisone replacement therapy was initiated at 6 days of life. Other pituitary axes were found to be normal, apart from transiently low FT4 (13.7 pmol/L) at the lower end of the normal range for age (11 to 32 pmol/L), with normal TSH levels. Persistent ACTH deficiency was subsequently confirmed under normoglycemic conditions. Pituitary MRI at 18 days of life showed normal anterior and posterior lobes and a normal pituitary stalk. Higher quality MR images were obtained 6 years later, with the same findings. As of last follow-up, at 7 years of age, the patient had had three episodes of adrenal insufficiency with hypoglycemia and seizures in the context of infectious diseases.

Six years after the index case, a younger brother was born post-term with forceps delivery, an APGAR score of 2, neonatal hypotonia, feeding difficulties, and hyponatremia (130 mmol/L). He partially recovered for a few days in the intensive care unit but then developed a fever due to orchitis, treated surgically. Hormone tests at 10 days or age revealed cortisol and ACTH levels below laboratory detection thresholds (respectively <3 nmol/L and <1.7 pmol/L), while other pituitary hormone levels were normal, leading to a diagnosis of IAD. Hydrocortisone treatment was started (15 mg/m2/day) and the patient rapidly improved. Clinical examination revealed grade II mitral insufficiency and scaphocephaly. Hydrocortisone treatment was continued, and as of last follow-up one year later, the patient was in good health, with no complications.

Targeted NGS sequencing revealed a homozygous c.840del *TBX19* mutation in both children. Their parents, who live in northern France, are apparently not consanguineous but both their families come from the same region in northern Morocco. As expected, both were found to be asymptomatic heterozygous carriers of the c.840del mutation. They have one other child (asymptomatic, not studied) and reported one miscarriage due to ectopic pregnancy.

## 4 Genomic data on the TBX19 mutation

**Figure 1:**
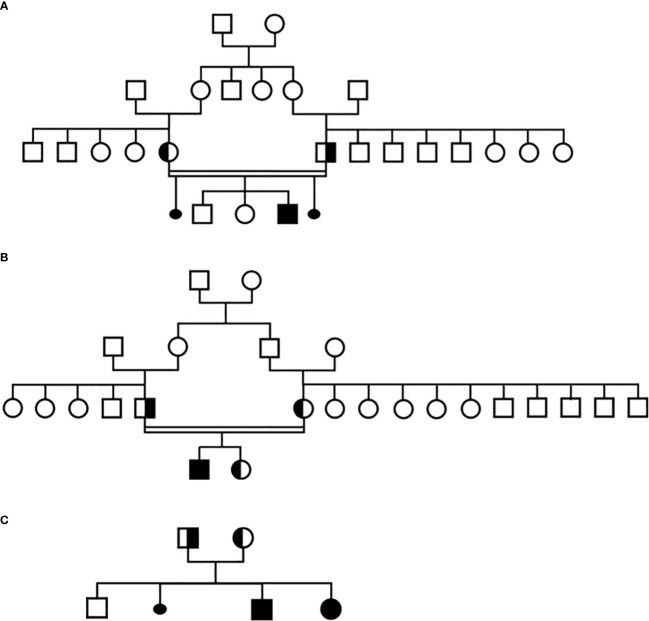
Pedigree of the three families in this study, **(A)** Family 1, **(B)** Family 2, **(C)** Family 3.

**Figure 2:**
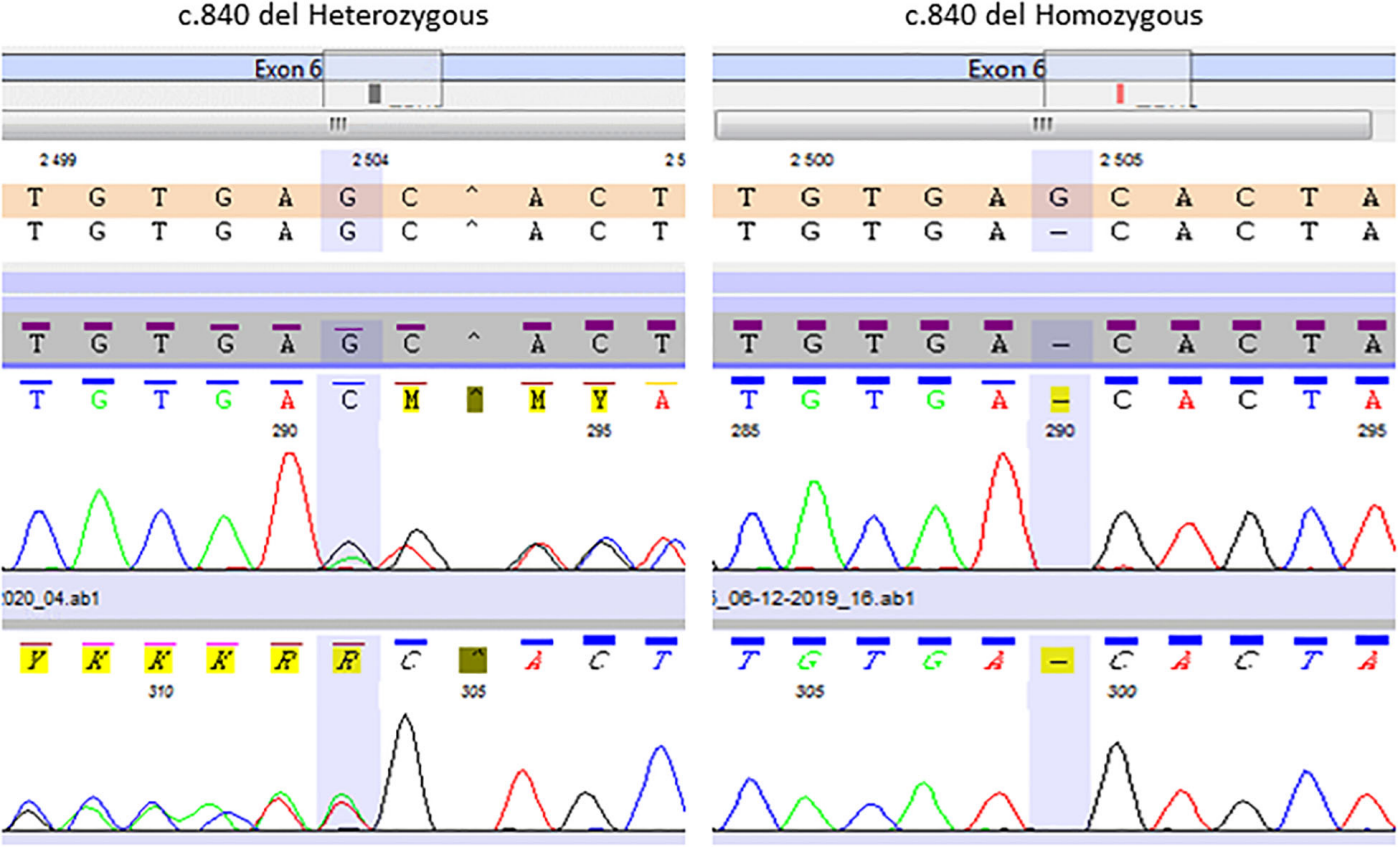
Representative Sanger chromatogram of the *TBX19* c.840del homozygous patients and heterozygous relatives.

**Figure 3:**
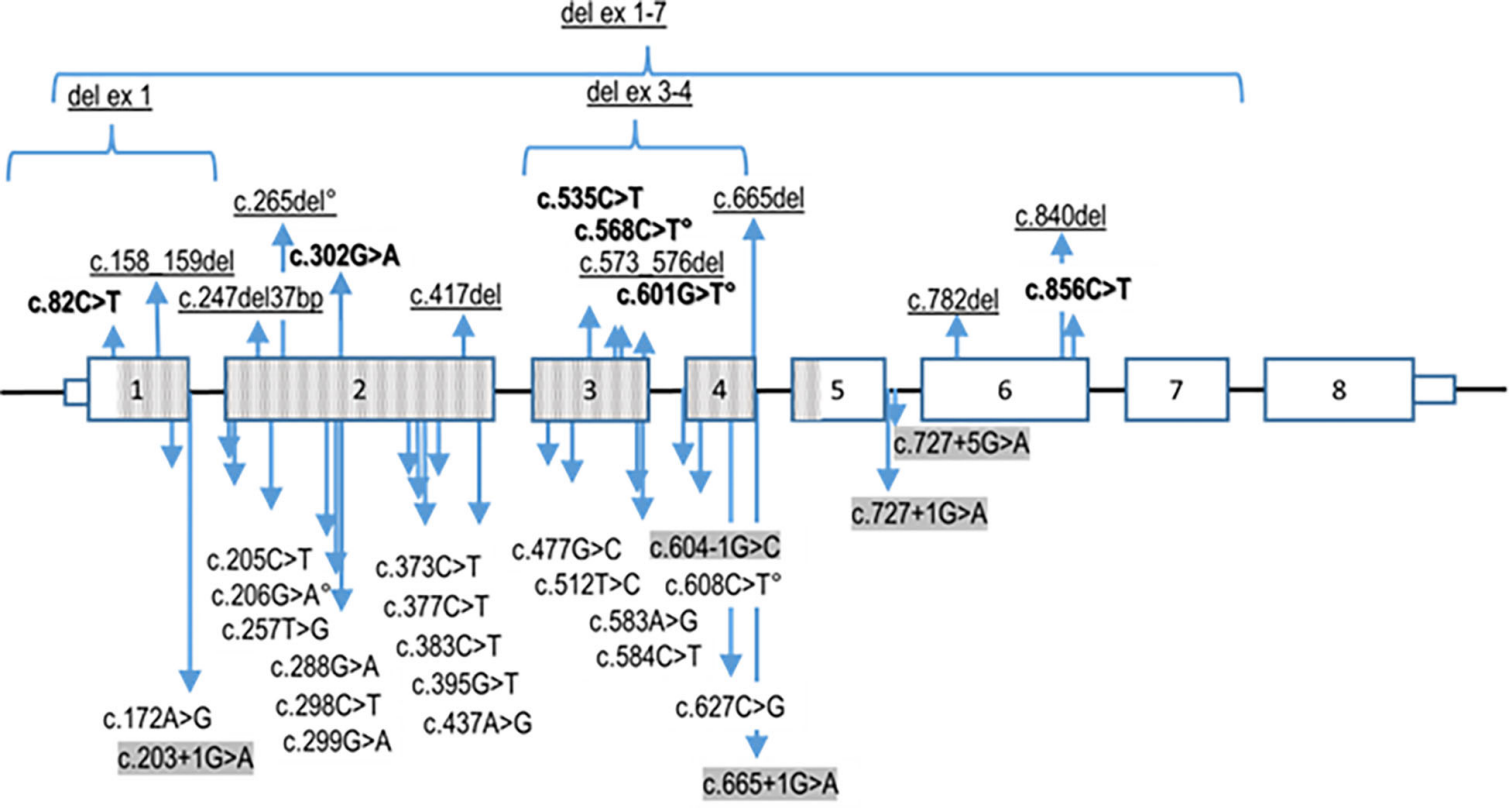
Summary of reported pathogenic *TBX19* variants. The nonsense variants (in bold), deletions of exons and indels (frameshift) variants (underligned) are listed above the *TBX19* gene schematic representation, whereas the missense and splice site (highlighted in grey) variants are presented underneath. Transcribed sequences are boxed. Small boxes indicate 5’- and 3’- untranslated regions. The bigger boxes represent the translated exons, while the hatched gray boxes indicates the region translated into the T-box protein domain. ° signals the variants reported on Clinvar without linked publication. Variants nomenclature is based on NM 005149.3 (MANE select) transcript.

The same molecular defect, a homozygous c.840del mutation, was identified by NGS in all four children with IAD, and all parents were heterozygous carriers (**Figure 1**). All the NGS results were confirmed by Sanger sequencing (heterozygous and homozygous example in **Figure 2**). The variant, which is not found in the general population gnomAD database (v2.1.1), is situated in exon 6 of the *TBX19* gene, in the 3’ region. Only 3 of the 39 previously described pathogenic variants of the gene are situated in this region (**Figure 3**).

The single-base deletion induces a frameshift with a premature nonsense codon, leading either to the synthesis of a truncated (so presumably nonfunctional) protein of 307 amino acids instead of 448 (p.(Glu280Asp fs*27)), or more probably, since the mutation is situated in exon 6 (out of 8), to nonsense- mediated decay and the complete absence of protein synthesis.

In terms of ACMG criteria, these observations—PVS1 (null variant in a gene where LOF is a known mechanism of IAD) plus PM2 (absent from controls in gnomAD V2.1.1 and dbSNP) plus PP4 (patient’s phenotype or family history is highly specific for a disease with a single genetic etiology, here, IAD) and/or PP1 (disease cosegregated with genotype)—classify the *TBX19* variant as pathogenic.

## 5 Discussion

This article describes four cases of IAD (from three families) due to the same previously undescribed pathogenic variant of *TBX19*. Our investigation of these cases was initiated following the discovery within 2 years of the same *TBX19* mutation associated with IAD in three different families with different names, from different regions of France. The link between these three families appeared after subsequent investigations. Suggestions of a common geographical origin from name research on genealogy websites were confirmed by patients when genetic testing results were delivered. All three families come from the same mountainous region of northern Morocco (Rif), strongly suggesting these cases are due to a founder effect.

Isolated adrenal deficiency was diagnosed in the neonatal period in two of the four children with this pathogenic *TBX19* variant, and in the two others at 2 and 5 months of life. Early onset IAD, diagnosed before 1 year of life is a typical feature of *TBX19* mutations (1). This point is noteworthy because four recently reported cases were diagnosed in older children, up to 5 years of age. However, the medical history of these patients (repeated hospitalizations and hydrocortisone treatment) suggests that IAD symptoms were already present before 1 year of age (5–7, 10).

Our patients’ phenotypes were very similar to those described for other *TBX19* mutations (1). Three patients had hypoglycemia, and this was only avoided in the fourth by the timely initiation of parenteral nutrition in the intensive care unit before IAD was diagnosed. Two patients also had hyponatremia, prolonged cholestatic jaundice, and or hypotonia, and one patient had seizures. Symptoms improved rapidly in all patients after hydrocortisone treatment was started, and with close monitoring and appropriate treatment adjustments, each of these children has shown normal growth, apparently good quality of life, and no signs of psychomotor retardation, over follow-up periods ranging from 7 months to 6 years. One patient nevertheless had three episodes of acute adrenal insufficiency during infections, probably because of a lack of timely dose adjustment. Avoiding decompensation and repeated severe hypoglycemic or hyponatremic episodes is essential for the outcomes of these children, especially in the first years of life. Indeed, one of the main consequences of undiagnosed adrenal crises and inadequately adjusted hydrocortisone treatment in IAD due to *TBX19* mutations is cognitive impairment (2, 4, 5, 20). Early diagnosis, close clinical monitoring in specialized centers and multiple therapeutic education sessions for parents are critical to avoid brain damage. The importance of parental education was highlighted in family 3, where better management of the second child led to clearly improved outcomes with fewer episodes of adrenal insufficiency.

No third trimester miscarriages or neonatal deaths were reported by these families, in contrast with previous studies in which they were reported in up to 25 percent of cases (1). However, two families reported first trimester miscarriages, including one due to ectopic pregnancy.

Since *TBX19* is only expressed in the pituitary gland, *TBX19* mutations are unlikely to lead to extrapituitary malformations. However, since autosomal recessive diseases are typically associated with consanguinity, other genetic defects cannot be excluded. Extrapituitary malformations, including a Chiari I malformation (1, 5) and microcephaly (5), have been reported in other cases of *TBX19* mutation. One of our patients had scaphocephaly (1/4) and two had cardiac valve defects (a bicuspid aortic valve, mitral regurgitation). These malformations are relatively frequent [1–2% for bicuspid aortic valve for instance (21)], and are therefore unlikely to be directly linked to the *TBX19* defect.

Our patients’ ACTH and cortisol levels were below laboratory thresholds in all cases, suggesting complete ACTH deficiency, as observed in the majority of previously described cases (1). The pituitary gland was considered normal on MRI in two of three patients and mildly hypoplastic in the third, in keeping with the results of a previous study in which all 11 investigated patients had normal pituitary MRI findings (1). Indeed, since *TBX19* is only required for the differentiation and proliferation of corticotropic cells, which represent roughly 15% of all pituitary cells (22), pituitary volume should only be marginally affected in TBX19-deficient patients.

All four patients had the same homozygous c.840del truncating mutation in *TBX19*. Most (22/39) of the *TBX19* gene variations described to date are truncating mutations, through various mechanisms (nonsense, frameshift, splice variants). It is noteworthy however, as shown in **Figure 2**, that almost all the described mutations (33/39) and all the missense mutations are located in the T box that encodes the DNA binding domain (1). The pathogenic variant reported here is only the sixth to have been found outside the T box domain, all of which are truncating mutations. The most frequently reported mutation (c.856C*>*T p.(Arg286*) (3, 7, 23), in line with our own experience (17), is located relatively close to the new c.840del mutation (p.Glu280Asp fs*27). Both mutations probably lead to an absence of protein expression through nonsense mediated decay (1, 4, 24).

The identification of a *TBX19* mutation in several members of a relatively small ethnic group, with strong arguments in favor of a founder effect, is unique. The only other *TBX19* mutation associated with a possible founder effect is the c.82C*>*T p.(Gln28Ter) mutation found almost exclusively in the Finnish population (43/45 of identified heterozygous individuals in gnomAD). Consanguinity increases the risk of autosomal recessive diseases and two of the three families in this study were consanguineous, in keeping with previous reports (5, 6, 20). Further studies are required to confirm the presence of a founder effect for the c.840del mutation and estimate the incidence of the variant in heterozygous state in the corresponding population.

For this purely autosomal recessive disease, the risk for heterozygous parents having a child with IAD is 25%. While measurement of estriol levels in pregnant women has been suggested as a means of diagnosing IAD before birth (22), prenatal diagnosis is not commonly used and access to a pediatric intensive care unit at birth is crucial in families with a history of IAD, to ensure optimal neonatal management.

## 6 Conclusion

In conclusion, we describe a new *TBX19* truncating mutation, identified in four children from three seemingly unrelated families and causing early onset IAD, a life-threatening neonatal condition. Our results highlight the importance of early diagnosis and treatment based on the observation of characteristic clinical signs, hypoglycemia and prolonged jaundice, sometimes appearing a few hours after birth and associated with complete IAD and normal pituitary MRI findings. Early diagnosis, timely treatment and preventive education allowed normal development, growth and quality of life in all four patients.

## Data availability statement

The data presented in the study are deposited in the ENA repository, accession number PRJEB59437.

## Ethics statement

The studies involving human participants were reviewed and approved by French Ministry of Health (Cellule de Bioe´thique, Direction generale de la Recherche et de l’Innovation, approval n° DC 2019-3568) and by the French data protection authority(Commission Nationale Informatique et Liberte´s, CNIL 16 September 2016, N° 1991429 v 0). Written informed consent to participate in this study was provided by the participants’ legal guardian/next of kin. Written informed consent was obtained from the individual(s), and minor(s)’ legal guardian/next of kin, for the publication of any potentially identifiable images or data included in this article.

## Author contributions

TC and AS analyzed the data, identified the pathogenic variant, and wrote the paper. AS and TB led the project and reviewed the manuscript. GM designed the primers and reviewed the manuscript. MP performed the technical NGS work. AB supervised NGS sequencing. RR, AC, IG, and FJ were the patients’ attending physicians, recorded their symptoms, medical history, and origin, and performed follow-up.
